# Breast Cancer Derived Extracellular Vesicles in Bone Metastasis Induction and Their Clinical Implications as Biomarkers

**DOI:** 10.3390/ijms21103573

**Published:** 2020-05-18

**Authors:** Simona Taverna, Ilaria Giusti, Sandra D’Ascenzo, Laura Pizzorno, Vincenza Dolo

**Affiliations:** 1 Institute for Biomedical Research and Innovation, National Research Council of Italy, 90146 Palermo, Italy; simonataverna.unipa@gmail.com; 2 Department of Life, Health and Environmental Sciences, University of L’Aquila, Via Vetoio-Coppito 2, 67100 L’Aquila, Italy; ilaria.giusti@univaq.it (I.G.); sandra.dascenzo@univaq.it (S.D.); 3 Breast Surgery Division, San Salvatore Hospital, 67100 L’Aquila, Italy; LPizzorno@asl1abruzzo.it

**Keywords:** EVs, extracellular vesicles, breast cancer, metastatic niche, bone metastasis, breast cancer biomarkers, liquid biopsy

## Abstract

Cancer incidence and mortality are rapidly growing worldwide. The main risk factors for cancer can be associated with aging as well as the growth of the population and socioeconomic condition. Breast cancer, a crucial public health problem, is the second cause of death among women. About 70% of patients with advanced breast cancer have bone metastases. In bone metastasis, cancer cells and osteoclasts form a vicious cycle: cancer cells promote osteoclast differentiation and activation that, in turn, induce cancer cell seeding and proliferation in the bone. Growing evidence shows that extracellular vesicles (EVs) play a key role in carcinogenesis, proliferation, pre-metastatic niche formation, angiogenesis, metastasis, and chemoresistance in several tumors, such as breast, lung, prostate, and liver cancer. Here, we discuss the role of EVs released by breast cancer cells, focusing on bone metastasis induction and their clinical implications as biomarkers.

## 1. Introduction

Breast cancer (BC) is the most prevalent malignancy in women worldwide. Over two-thirds of BC cases are diagnosed in women over 50 years old, with an incidence that seems to be higher for wealthy women. On the contrary, in women under 50, the number of diagnosed cancers is double in developing countries compared to developed states [1].

The mortality associated with this tumor is decreasing in the last years, especially in those countries where women adhere to recommendations for routine screening by breast echography and mammography [1]. Despite this, the International Agency for Research on Cancer (IARC) reported 626,000 deaths worldwide in 2018 [2,3]. One of the major causes of therapeutic failures is the extensive heterogeneity of breast cancer subtypes and metastatic dissemination, with different clinical features.

**Figure 1:**
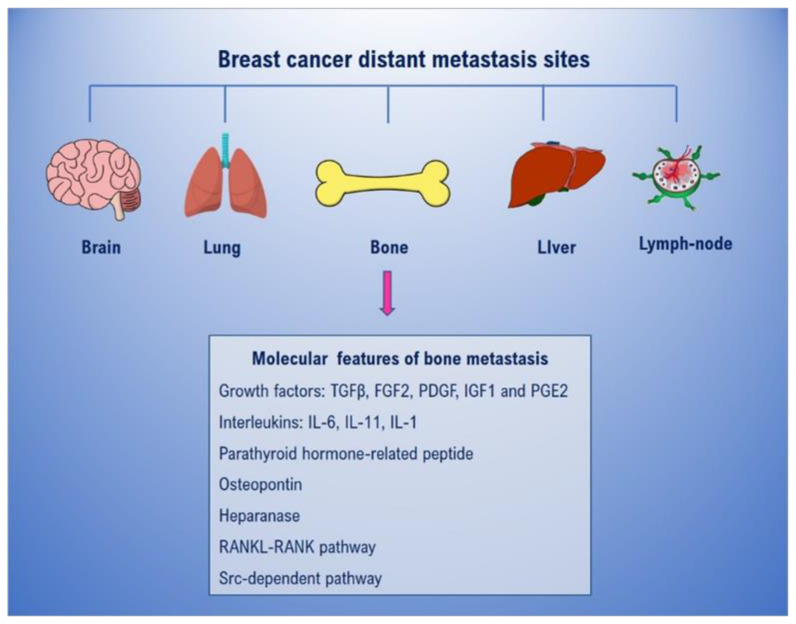
Breast cancer distant metastasis sites and molecular features of bone metastasis.

BC is divided into four principal categories based on presence or absence of molecular markers for estrogen, progesterone receptors, and human epidermal growth factor 2 (ERBB2). The subclasses are: luminal A, luminal B, HER2 positive, and triple-negative (TNBC) [4]. TNBC are tumors lacking all the standard molecular markers. Although more than 90% of breast cancers are not metastatic at the time of diagnosis [5], the preferential metastasis sites are bone, lung, brain, lymph nodes, and liver (Figure 1) [6,7].

Bone metastases have strong consequences in BC patients’ lives, causing severe pain, bone fractures, hypercalcemia, spinal cord compression, and other nerve compression syndromes [8,9,10]. Bone pain of inflammatory or mechanical origin, indeed, is one of the most frequent complications of bone metastasis in cancer patients. In first stages it is usually reported as a dull pain but, as cancer grows, may become more intense. Painful bone fractures, mainly in proximal parts of long bones (femur in over half of cases), but also in ribs or vertebras, have also been reported in a small percentage of patients with bone metastases [9,10]. Hypercalcemia is another common paraneoplastic syndrome, partly due to the bone resorption caused by osteolytic metastasis. A further severe clinical consequence of bone metastasis is the “metastatic epidural spinal cord compression” which is cause of intense pain, motor weakness and sensory deficits [9].

Several risk factors are associated with BC onsets such as hormonal, reproductive and menstrual history, age, ethnicity, hereditary factors, and other modifiable factors such as eating habits, physical inactivity, or exogenous hormones [1]. Genetic background increases the risk to develop BC. About 10–30% of breast cancers are related to hereditary factors. Among these cases, 5–10% are linked with strong hereditary factors and about 5% have mutations in high-penetrant genes, such as BRCA1 and 2. These genes are regulators of DNA repair, transcription, and cell cycle. Frequently, when mutated, they are associated with a higher predisposition to several cancers, included BC. It was described that 60% of hereditary breast cancers are related to germline mutations in BRCA genes. BRCA mutations, indeed, may be inherited (germline) or arise de novo as a result of combinatorial genetic and environmental factors (somatic) [1].

Some single nucleotide polymorphisms (SNPs) have been also related to BC risk. SNP is the substitution of a single nucleotide occurring at a specific gene’s position. SNPs related to BC risk have been detected on *XRCC2* and *XRCC3* genes. These genes encode for proteins that participate in homologous recombination of DNA double-strand breaks maintaining chromosome stability [11]. Some other common SNPs, associated with BC risk, affect *CASP8* gene, encoding for caspase 8, a protease with an important role in apoptosis initiation, the programmed cell death that usually follows DNA damage [12].

Many BC patients die from distant metastases. BC cells metastasize to specific organs; this process is known as organotropic metastasis [13]. Metastatic organotropism is a non-random process regulated by several factors in which tumor mass and host microenvironment contribute to the premetastatic niche (PMN) formation [14]. This complex network involves several cytotypes, soluble factors, and extracellular vesicles (EVs) [15].

EVs derived from the primary tumor, in fact, are potential mediators for PMN formation. EVs released by BC cells shuttle several molecules involved in bone metastasis induction.

In this review, we focus on the role of EVs released by BC cells in bone metastasis and their clinical implications as biomarkers.

## 2. Breast Cancer and Bone Metastasis

Solid cancers frequently metastasize to bone, as arises in about 70% of lung, prostate, and breast cancers. In patients with BC, the skeleton is the most frequent metastasis site [16]. Bone metastasis is a frequent, wasting, and incurable breast cancer complication [13]. In most cases, we have observed bone metastases in BC patients with large neoplasms already at the moment of diagnosis but also, in some cases, BC patients with small tumors who have bone metastases diagnosed during preoperative staging or even the appearance of bone metastasis in BC patients underwent surgery 15–20 years earlier (personal observations).

Physiological bone remodeling is the result of a perfect balance between osteogenic functions of osteoblasts and osteolytic activity of osteoclasts. This process allows for constant bone regeneration, mediated by systemic and paracrine factors that regulate osteoblast and osteoclast functions.

Bone tissue mainly contains three cytotypes: osteoblasts, osteoclasts, and osteocytes. Osteoblasts originate from pluripotent mesenchymal stem cell, secrete matrix and promote bone formation. Osteoclasts are multinucleated macrophages derived from monocytes that degrade bone matrix activating specific enzymes and generating acid microenvironment. Osteocytes derive from osteoblasts once they have been embedded in mineralizing bone [17].

Bone is a favorable site of tumor metastasis since it is a vascular organ, which provides nutrients sufficient for tumor cell survival. Moreover, low pH, intramedullary hypoxia, and high extracellular calcium concentration induce tumor engraftment [13].

Metastatic BC cells move from breast tissue, extravasate from capillaries to bone marrow and acquire bone cell-like properties by osteo-mimicry that improves homing in the bone. Thus, these circulating tumor cells (CTCs) adhere to bone surface and the bone, in turn, supports CTCs to proliferate and survive, modulating bone microenvironment [18]: the interactions between CTCs and bone components mediate tumour cell anchorage, survival, micrometastasis, and osseous colonization.

**Figure 2:**
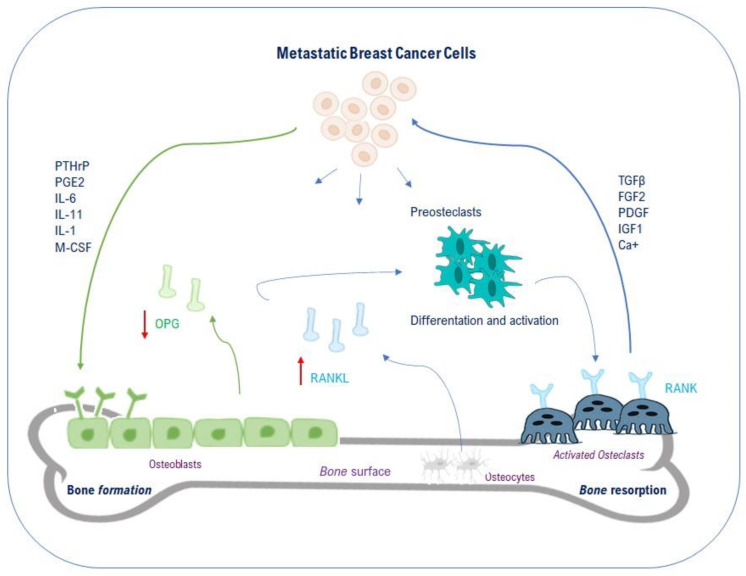
Schematic representation of vicious cycle between cancer cells and bone. Cancer cells secrete soluble factors (PTHrP, PGE2, ILs, M-CSF), which act on osteoblasts and osteoclasts in bone metastatic site. RANKL production is increased and OPG secretion is decreased from osteoblasts; OPG in physiological conditions acts as a decoy receptor binding the excess of RANKL. The up-regulated RANKL interacts with RANK receptor on preosteoclast. Preosteoclasts respond with their differentiation and osteolytic activation: PDGFs, BMPs, TGF-β, IGF1, and calcium ions released by degraded bone matrix can further enhance tumor cells survival. These cells produce more PTHrP which, in turn, reinforces bone resorption. Red arrows indicate the increase or decrease of molecules’ levels. Blue arrows suggest the relationship between different components of the vicious cycle.

Once in the bone, in fact, BC cells release several factors such as interleukins, osteopontin, parathyroid hormone-related peptide (PTHrP), prostaglandin E2, and heparanase that can induce osteoclasts activation and bone resorption. In particular, PTHrP released by BC cells binds to osteoblasts via its receptor and then induces Receptor-Activator-of-Nuclear-factor-Kappa-B-Ligand (RANKL) up-regulation and Osteoprotegerin (OPG) down-regulation (in physiological conditions OPG acts as a decoy receptor binding the excess of RANKL). RANKL overexpressed by activated osteoblasts binds to its receptor RANK on preosteoclasts. Then, the activation of the RANKL-RANK signaling pathway induces the differentiation of preosteoclasts into activated osteoclasts and leads to bone resorption. Successively, activated osteoclasts degrade bone matrix by releasing proteinases and hydrogen ions to create the acid environment [19,20,21,22]. Furthermore, resorbed bone secretes specific growth factors, such as IGF1, PDGF, TGFβ, and calcium, that enhance tumor proliferation in osseous [18]. Overall, the relationship between bone resorption and tumor growth forms a “vicious cycle” (Figure 2).

The majority of patients present CTCs at diagnosis, even without evidence of clinical osseous metastatic disease [23].

Bone metastasis can result in pathological bone loss (osteolysis) or bone formation (osteoblastic lesions). Up to 75% of stage IV BC patients develop skeletal metastases that can be osteolytic (where bone resorption occurs at a rate faster than bone deposition) or osteoblastic (where there is an increase in bone deposition) or both [24]. Most patients with BC have mainly osteolytic lesions, while 15–20% of them have osteoblastic lesions.

Whilst the role of osteoclasts in bone metastasis has long been known, osteoblasts’ one has being revealed in the last few years. Several papers confirmed that osteoblasts’ biology is deeply altered by BC cells. They no longer differentiate nor deposit new matrix but, instead, are diverted to produce cytokines and growth factors (such as IL-6, IL-8, MCP-1, GRO-alpha, and VEGF) that support cancer cell proliferation. Moreover, the observation that BC cells frequently are found in bone areas enriched in osteoblasts suggest they could be involved in cancer cells homing to bone, facilitating tumor cells’ escape from dormancy [22,25]. Despite current anti-osteolytic therapies delaying the deleterious process of bone resorption, alteration of bone remodeling increases osteoclast activity through the change of normal balance between RANKL and OPG. This bone damaging evolution induces the aforementioned “vicious cycle” in which growth factors released by osteoclasts can stimulate tumour growth and other molecules released by cancer cells to enhance, in turn, osteoclast differentiation [24].

Many studies aim to identify new therapeutic targets. With RANKL/RANK being fundamental in the vicious cycle of bone metastasis in BC, a wide variety of newly considered therapeutic strategies aim to intervene, both directly or indirectly in this cross-talk. Numerous drugs have been and are being tested in this regard, included bisphosphonates, recombinant OPG-Fc, and anti-RANK-L antibodies [18,26].

A recent study reports that BSA-coated gold cluster might prevent breast cancer bone metastasis by suppressing tumor-induced osteoclastogenesis. Zhang et al. demonstrate that gold clusters suppress migration, invasion, and colony formation of MDA-MB-231 cells in a dose-dependent manner and RANKL-induced osteoclast formation from bone marrow-derived mononuclear (BMM) cells in vitro. The gold clusters inhibit osteolysis-related factors expression in MDA-MB-231 cells that, in turn, inhibit NF-κB pathway activation in BMM cells. Furthermore, a mouse model indicates that gold clusters can reduce osteolysis induced by breast cancer cells in vivo. Overall, gold clusters may be useful as new therapeutic agents for avoiding BC bone metastasis and secondary osteolysis [27].

As aforementioned, several cytotypes and soluble factors can synergistically induce bone metastasis in breast cancers; the network of BC cells, osteoclasts, osteoblasts, and released cytokines caused a vicious cycle that exacerbates cancer progression [28,29]. Moreover, calcium ions are released by bones during bone metastasis causing hypercalcemia, but this process is not well-known in bone metastasis modulation [30]. Calcium ions act as a second messenger with different effects on tumor cells, osteoclasts, and osteoblasts, fluxing dynamically among extracellular calcium channels.

Calcium homeostasis is a balance between intracellular calcium storages and cytosolic calcium signals. The alteration of this process leads to calcium ions release from bone [31], inducing a vicious cycle between breast cancer cells, osteoclasts, and osteoblasts that enhance PMN formation. The four major calcium routes are TRPs, VGCCs, SOCE, and P2Xs which play important roles in tumor cell proliferation, survival, migration, metastasis, and modulation of osteoclast differentiation and activation [31]. Understanding calcium homeostasis role in bone metastasis mediated by calcium channels could provide potential new therapeutic options in combination with well-known chemotherapeutic treatments for cancer management.

A new study shows that Olaparib might increase breast cancer bone metastasis through PARP2 (poly ADP-ribose polymerase), but not PARP1, particularly in myeloid lineage and not in cancer cells [32]. Olaparib is a PARP1 and PARP2 dual inhibitor recently FDA approved for advanced breast cancer treatment; its effects on bone metastasis are unknown. PARP inhibitors cause synthetic lethality in BRCA-mutated cancer cells from defective DNA damage repair [33]. Olaparib treatment or PARP1/2 deletion promotes osteoclast differentiation and bone loss. Myeloid deletion of PARP2, but not PARP1, increases immature myeloid cells population in bone marrow, and impairs chemokines expression such as CCL3 (chemokine (C-C motif) ligand 3), through enhancing β-catenin transcriptional repression. Altered CCL3 expression induces an immune-suppressive condition by modulating T cell subpopulations. Many BRCA-mutated cancer patients do not respond to PARP inhibitor treatment. Acquired PARP inhibitor resistance is often observed due to different mechanisms, including BRCA function restoration by secondary mutations [34]. Since PARP1/2 dual inhibitors might exacerbate bone metastasis, new biomarkers to early predict the sensitivity of PARP inhibitors and long-term clinical outcome are necessary. These data suggest a careful evaluation of PARP inhibitors on bone metastasis and suggest co-treatment with CCL3, β-catenin inhibitors, anti-RANKL, or bisphosphonates as a potential combination therapy for PARP inhibitors [32].

## 3. Extracellular Vesicles and Cancer

Extracellular vesicles (EVs) have been described for the first time as devices to eliminate “cellular garbage” [35]. Nowadays, it is well known, instead, that EVs have a key role in intercellular communication: they are small vesicles released constitutively by all cytotypes of mammalian individuals and lower eukaryotic and prokaryotic organisms, in both physiological and pathological conditions.

The scientific community distinguishes two major populations of EVs basing on their cell origin: microvesicles (MVs) and exosomes (EXOs) [36,37]. MVs, with a diameter ranging between 100–1000 nm, are shed directly by the plasma membrane. EXOs are nano-sized spherical vesicles (30–150 nm in diameter) originating from the endo-lysosomal compartment after fusion of multivesicular bodies with the plasma membrane and the release of their intraluminal vesicles into the extracellular milieu.

Recently, Exocarta and Vesiclepedia databases included proteins, lipids, miRNAs, and mRNAs, as contained within EVs collected from 1254 published studies (http://microvesicles.org) [38]. These databases indicate the potential targets that can be modulated by EVs and highlight EVs’ importance in the oncology field.

EVs are contained in several biological fluids such as blood, saliva, urine, amniotic and cerebrospinal fluid, breast milk, seminal fluid, and malignant effusion [39,40,41,42,43].

It was demonstrated that cancer cells release large amounts of EVs, compared with normal cells, to promote cancer progression [44].

The plasma of patients with BC contains higher concentrations of EVs than plasma of healthy controls: in particular, an increase of EXO amount in plasma of TNBC patients respect to patients with other subclasses of BC was reported [45,46].

As mentioned, EVs contain several biomolecules, such as lipids, proteins, DNA, and RNA (mRNA, miRNA, lncRNA) that mirrored the cell and tissue of origin [47,48,49,50,51,52,53]. Recently, circRNAs have also been detected in EVs and can be considered new non-invasive cancer biomarkers [54]. Several proteomic studies have demonstrated that EVs contain membrane, cytosolic and cytoskeletal proteins, adhesion molecules, integrins, enzymes, signaling molecules, and tumor-specific proteins. Among EV-proteins, heat shock proteins, and tetraspanins are the most conserved molecules [55,56,57,58]. Recently, it was reported the presence of Tetraspanin 8 (Tspan8) in breast primary tumor and metastases indicating its role as a regulator of cell behavior and EV release in breast cancer. Tspan8 can mediate up-regulation of E-cadherin and down-regulation of Twist, p120-catenin, and β-catenin target genes inducing a cell phenotype switch, similar to the mesenchymal–epithelial transition. Moreover, Tspan8+ cells shown an increase of cell-cell adhesion, and decrease of motility and sensitivity to irradiation. Tspan8, as a regulator of EVs content and function, can mediate EV amount increase in cell culture and in the circulation in tumor-bearing animals. These results indicate a potential role of Tspan8 as a therapeutic target and as a non-invasive biomarker [59].

EV-proteins maintain their biological activities including pathway activation, protein cleavage, and antigen presentation. As aforementioned, EVs contain nucleic acids, such as mRNAs that can be translated in target cells, and microRNAs that mediate RNA-silencing, thus allowing dynamic regulation of gene expression. It was also reported that EVs contain DNA which would allow the identification of gene mutational status [60,61].

Different ways of interaction between EVs and target cells have been described. EVs can be internalized: by their binding to surface receptors that trigger intracellular signaling; by fusion to plasma membranes of target cells. By breaching and releasing of their contents into extracellular space with a pH-dependent mechanism [62,63]. Recent studies also indicate that EVs uptake is mediated by micropinocytosis and clathrin-independent endocytosis [64]. Moreover, EVs can be also internalized: (1) surfing on filopodia to enter cells at endocytic hot spots, traffic within endosomes in order to arrive at the endoplasmatic reticulum [65], or (2) through filopodia extensions from cell membranes [66].

EVs are widely involved in intercellular communication between cancer cells and tumor microenvironment network. Several studies indicate that EVs play a key role in tumorigenesis, tumor growth, angiogenesis, tumor immune modulation, drug resistance, epithelial–mesenchymal transition (EMT), preparation of a PMN, and metastasis [67,68,69,70,71,72,73,74].

Several bioactive molecules contained in EVs can cooperate in a complex network of different cytotypes to induce tumor progression and metastasis. For example, it was described that EVs can transport mutated DNA that, interacting with target cells, induces a phenotype switch. Specifically, it was reported that lung cancer EVs shuttle EGFR mutated genes, which induces the target cells to become resistant to TK inhibitor, the classical inhibitor of EGFRs [75]. BC cells that harbor KRAS mutations secrete EXOs that are enriched in KRAS and EGFR ligands, which enhances the invasiveness of neighboring recipient cells [76].

BC was one of the earliest tumors where EVs-associated drug resistance was investigated [74]. Other that the well-known ability of cells to enclose drugs within EVs to eject them outside the cell (preventing the drug from reaching the effective dose) [77]. It has also been highlighted that EVs released by drug-resistant cells can be used to transfer the resistance to drug-sensitive cells by means of proteins, miRNAs or mRNAs [78,79,80,81]. Drug resistance is not only mediated by molecules transfer but it seems that EVs itself could affect some drugs’ efficacy. For example, EVs from HER2-positive cells, but not from HER2-negative ones, bound to Trastuzumab inhibiting its anti-proliferation activity [82].

Furthermore, EVs carry bioactive molecules through which cancer cells can communicate and re-program the immune system. Recently, it was reported that EVs isolated from plasma of patients with different tumors carry programmed death-ligand 1 (PD-L1) and PD-1 and these vesicles seem to have immunosuppressive properties [83]. In metastatic melanomas, EVs carry PD-L1 on their surface, which suppresses the function of CD8 T cells and facilitates tumor growth [19].

## 4. Role of Extracellular Vesicles in Bone Metastasis Induction

Tumor cells release EVs that influence cell behavior in the primary tumor microenvironment and distal sites. Although several mechanisms are described for tumor progression and metastatic organotropism [84], EVs role in these processes is being clarified [85].

Experimental evidences indicate that EVs are internalized by specific cells to prepare a permissive microenvironment and initiate PMN in distant sites [86]. PMN formation includes vascular remodeling, immune modulation, and recruitment of non-immune stromal cells, metabolic reprogramming, and determination of organotropism of the primary tumor to promote metastasis at specific distant sites. The organotropism can be considered as crosstalk between primary tumor cells and metastatic site network [87]. EVs participate to tumor microenvironment reprogramming, representing the “soil” at distant metastatic sites that determine cancer cell growth [88].

Recently, EVs role in bone metastasis was described for different cancer types. It was described that EXOs released by multiple myeloma positively modulate pre-osteoclast migration, increasing CXCR4 expression, and activating a survival pathway. This process induces osteoclasts’ activation and differentiation supporting osteolytic metastasis [89].

Moreover, EXOs released in vitro by non-small cell lung cancer (NSCLC) cells were internalized by pre-osteoclast cells, inducing osteoclast differentiation. NSCLC-EXOs contain Amphiregulin (AREG), an important EGFR ligand that induces EGFR pathway activation in pre-osteoclasts which, in turn, causes increased expression of RANKL. RANKL induces proteolytic enzyme expression, well-known markers of osteoclastogenesis, activating the vicious cycle in osteolytic bone metastasis. It was also described that EXOs derived from NSCLC patients’ plasma contained AREG which induced osteoclast differentiation in human pre-osteoclasts [90].

Osteoblastic lesions could also be induced by tumor EVs [91]; it has been demonstrated, for example, that both prostate and BC release miR-940 by EXOs that induced, in bone microenvironment, extensive osteoblastic lesions in vivo [92]. Similarly, miR-141-3p loaded in prostate cancer cells EXOs promotes osteoblastic metastasis [93].

The role of EVs released by BC cells in bone metastasis induction will be described below in a specific paragraph.

## 5. Role of Breast Cancer Extracellular Vesicles in Bone Metastasis Induction

**Figure 3:**
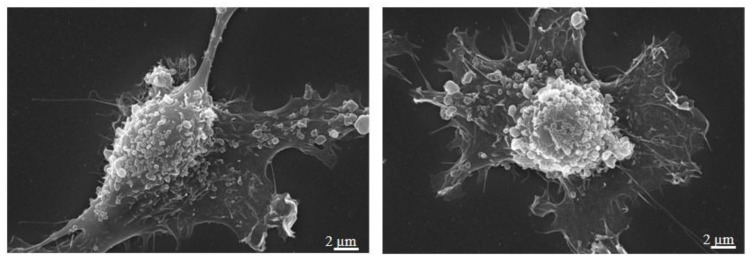
Scanning Electron Microscopy of MDA-MB-231 cells showing EVs release from cell surface. Magnification 10,000×; scale bar 2 µm (personal images).

As for many tumour cells, breast cancer cells release a great number of heterogeneous-in-size vesicles from the cell surface (Figure 3).

**Figure 4:**
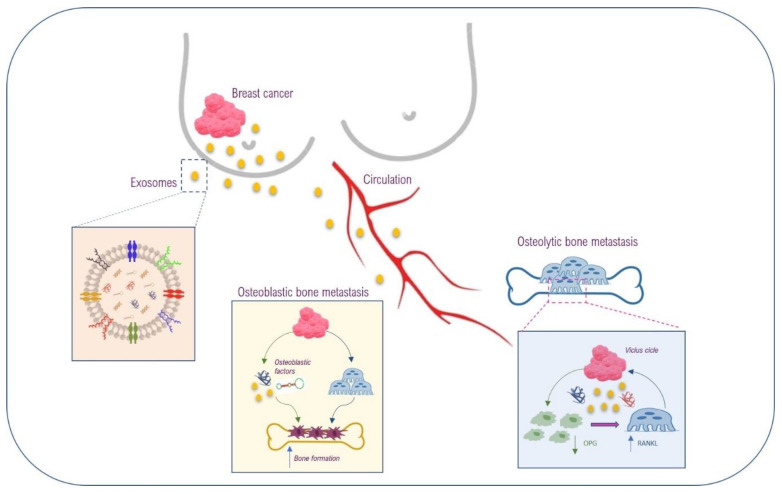
Schematic representation of EVs involvement in the communication between breast cancer cells and bone/bone marrow niche. BC cells of primary tumor release both soluble factors and EVs containing lipids, proteins, DNA, mRNAs and microRNAs (schematically represented in the insert on the left); CTCs also move from primary tumors towards bone. Both EVs and CTCs arrive to bone tissue through circulation. Soluble and EVs-associated factors can modulate bone microenvironment supporting BC cells in osteoblastic or osteolytic bone metastasis induction.

A large number of in vitro and preclinical in vivo studies, as well as clinical evaluations, have contributed to unravel the EVs role in breast cancer biology, suggesting that EVs could also be involved in the communication between BC cells and bone/bone marrow niche (Figure 4).

Several papers addressed the issue of EVs-associated molecules released by BC cells and their role in bone biology.

Some of these studies highlight EVs involvement in BC cells dormancy in the bone marrow. Cancer dormancy is generally defined as the arrest of tumor growth in the primary or the metastatic site, due to a quiescent state of cancer cells [94]. It has a huge meaning from a clinical point of view, since quiescent or dormant cells are undetectable and often resistant to traditional chemotherapeutics, being their proliferation slowed [95]. Breast cancer cells and mesenchymal stem cells in bone marrow communicate through EXOs: cancer cells stimulate, in mesenchymal stem cells, the release of miR-222/223 containing-EXOs that, in turn, promote the quiescence in a subset of cancer cells, also affecting the drug resistance [96]. Similar effects had already been reported for miR-23b expression: this miRNA is associated with EXOs released from bone marrow mesenchymal stem cells and promotes dormancy in breast cancer cells driving them to enter in G0 phase, confirming that exosomal transfer of specific miRNAs can deeply affect cancer cell dormancy in the metastatic niche [95].

As mentioned, BC has bone district as a preferential site of metastasis (along with brain, liver, and lung) and the EVs role in this process has just begun to be revealed [97]. Metastasis requires for cancer cells in the primary tumor to acquire invasive features, but also the generation of PMN whose formation relies on molecules released by tumor cells that increase the chance for arriving tumor cells to successfully “seed” the “soil” [98]. These tumor released-molecules can be soluble ones or EVs-associated.

Several EV-associated miRNAs have been demonstrated to contribute to metastatic processes in breast cancer. BC cells secrete EVs-associated miR-105 targeting endothelial cells and weakening the integrity of endothelium, the natural barrier against metastasis, by disrupting its tight junctions [99]. Similar mechanisms have been also obtained for brain metastasis: EV miR-181c, for example, has been reported for its ability to disrupt the blood brain barrier to facilitate the cell passage and playing a role in BC cells metastasis into brain [100]. Once in the brain, moreover, breast cancer cells survival is increased by EV-encapsulated miR-19a released by astrocytes that acts by decreasing PTEN expression [101]. It can only be speculated that similar mechanisms could also be found in bone metastases but has not yet been demonstrated.

EV-contained miR-122 released by BC cells can suppress glucose uptake by non-tumor cells in the pre-metastatic niche, thus reprogram the energy metabolism (an emerging hallmark of cancer) in a way that leaves more glucose available for metastatic cancer cells [102]. MiR-940–overexpression induced in the BC cell line MDA-MB-231 has been shown to induce extensive osteoblastic lesions in in vivo mouse models by facilitating the osteogenic differentiation of host mesenchymal cells. The authors demonstrated that the transfer of EXOs from cancer to stromal cells was responsible for the osteoblastic lesions’ induction [92].

Thus, EVs-associated miRNAs are currently the molecules that arouse greater interest due to their supposed role in breast cancer metastasis to bones (Table 1).

Even if not specifically demonstrated for bone metastasis, EXOs from highly metastatic BC cells potentially contribute to creating a premetastatic niche also able to promote metastasis by altering immune cells’ activity: EXOs were able to suppress CD8 and CD4 T-cells proliferation and reduce the NK cytotoxic activity against target tumour cells [103].

## 6. Breast Cancer EVs as Biomarkers in Clinical Applications

Breast cancer EVs are considered not only to identify possible targets in their cargo of therapeutic interest; they may also represent a tool for cancer treatment since they are good candidates as biomarkers for cancer diagnosis and could be used as drug-carrier for therapy [104].

The early diagnosis and monitoring of cancers through blood tests have been for many years the main goal of medical diagnostics. In this regard, research has identified a powerful tool in “liquid biopsy”. Liquid biopsy is a term that refers to the non-invasive analysis of tumor-derived material circulating into blood, such as circulating tumor DNA (ctDNA), CTCs, and EVs. In the last years, beside to these classical targets of liquid biopsies new constituents have been considered, included circulating tumor RNA (ctRNA) and tumor-educated platelets (TEPs) [105].

Liquid biopsy could overcome the typical limitations of tissue biopsy, that are the invasive procedure associated with patient risk and pain, high procedural costs and limits in sample preparation, sensitivity, and accuracy. Moreover, it also has many other advantages compared to tissue biopsies, such as the ability to represent tumor heterogeneity and the possibility to be repeated over time to monitor tumor progression or response to treatments [105].

EVs are released into biological fluids in large amounts from tumor cells thus representing a simple way of access to biomarkers. The possibility to use EVs-associated molecules as cancer biomarkers is justified by the fact that molecular cargo mirrors the cell of origin, thus being representative of the parental cells. EVs ensure the stability of their cargo since it is protected by their outer lipid membrane. Their unique composition and their long in vivo stability strongly support the belief that EVs fully meet the requirements to be useful biomarkers for various malignancies [106]. It must be added that it has also been suggested that some EV-associated molecules may be predictive of response to treatment or be useful as diagnostic tool for minimal residual disease detection, further paving the way to a broad clinical application [107,108].

Several studies also confirm in vivo what was already known from in vitro observation: tumor cells release far more EVs than normal counterpart [108,109,110]. Thus, quantitative or qualitative EVs modifications could provide useful information [111].

As for many other tumors [112,113,114,115,116,117,118,119,120,121], for breast cancer, the possibility to use EVs or EVs-associated molecules as biomarkers has been widely considered too [122,123,124,125,126]. Many molecules, of different molecular identity, have been taken into consideration as biomarkers: nucleic acids, proteins, lipids, metabolites.

Many papers focused on nucleic acids, particularly miRNAs. miRNAs, in fact, are considered promising candidate biomarkers being widely involved in cancer progression and characterized by cancer-specific expression profiles. Several in vitro and in vivo studies revealed the role of breast cancer EVs-miRNAs in modulating cell proliferation and apoptosis, as well as the metastatic process or the drug resistance/sensitivity; they also modulate endothelial cells biology and induce fibroblasts activation into CAFs (cancer-associated fibroblasts), deeply affecting the tumor microenvironment [127,128]. Clinical settings also support the potential role of EVs as promising biomarkers for both the early diagnosis of breast cancers and for the therapeutic effect, as well as the patient’s outcome evaluation [123].

MiR-939, characterized by a higher expression in human breast cancer compared to normal mammary tissues, was one of the evaluated miRNAs. An in vitro study shows how it targets the VE-cadherin (a component of adherent junctions in endothelial cells) decreasing the monolayer integrity and thus resulting, supposedly, in vessel permeability alteration. miR-939 resulted to be associated with EXOs released by BC cells and to be responsible for an increased tumor cell trans-endothelial migration. The authors also analyzed miR-939 levels in breast cancer patients founding that it was highly expressed in the basal-like tumor subtypes and triple-negative breast cancers and identifying it as a negative prognostic factor for disease-free survival. So, the paper suggests an association between miR-939 levels and a worse prognosis in TNBC, possibly supported by its role in the disruption of endothelium integrity that could favor tumor spread by hematogenous mechanism [129].

Hannafon et al. showed that many miRNAs were selectively associated with EXOs released from cell lines of normal mammary epithelial and breast cancer (MCF10A, MCF7, MDA-MB-231). Some of those miRNAs were enriched in EVs isolated from breast cancer cells compared to normal epithelial cells (10 miRNAs in MCF7 and 132 in MDA-MB-231), with miR-1246 and miR-21 being the most expressed in EXOs released by MCF7 and MDA-MB-231 cells respectively and, thus, considered a good candidate as circulating biomarker. Those two miRNAs were also analyzed in human blood and found to be highly enriched in EXOs isolated from plasma of patients with BC when compared to those from healthy subjects with no history of breast cancer. The authors also highlighted that the combined use of both miRNAs was a better indicator than their individual level expression, suggesting that analysis of specific miRNA, or maybe a combination of miRNAs, could be a new strategy to detect the presence of breast cancer [130].

EVs-associated mRNAs have also been taken into consideration in a few studies, suggesting the potential of their use for therapy monitoring [131].

Many other papers, instead, considered the protein cargo of EVs. Proteomic analysis of circulating EVs in breast cancer patients and healthy subjects revealed different protein content. Interestingly, different tumor stages (III and IV) were associated to different profiles, revealing a unique and distinctive cargo. Further analysis focusing on three molecules (PAI-1, ADAM12, and β-catenin), already known for their association to BC clinical outcome, showed that their levels are correlated with the disease stage (i.e., more abundant in advanced stages). Another interesting data is that the plasma EVs contained ER-α, PR, and HER-2 (the three main prognostic markers for breast cancer), and that their levels correlated with their expression measured by immunohistochemistry in the corresponding tumors. Overall, EVs’ ability to carry BC markers and to exhibit protein levels modulated according to the disease state strongly supports the hypothesis that they may represent a non-invasive source of diagnostic biomarkers for breast cancer [132].

The same indications come from another study that shows breast cancer EVs proteome profile. EVs isolated both from in vitro cell lines and plasma of BC patients allow BC subtyping [133].

Chen et al. assessed the state of protein phosphorylation in EVs from human plasma, comparing breast cancer patients to a healthy status. The phosphorylation status of proteins, indeed, is one of the most important regulatory mechanisms to control many aspects of cellular functions and it could be related to disease status. The authors were able to identify, in plasma isolated EVs, 144 phosphoproteins whose levels were higher in patients diagnosed with breast cancer compared to healthy individuals, suggesting that the use of EVs-associated phosphoproteins could be feasible and may affect the approach to cancer screening and monitoring [134].

The proteoglycan glypican-1 (GPC1) is another molecule, specifically enriched in cancer cell-derived EXOs, that has been assessed as a potential biomarker. Even if results mainly support its use as a diagnostic and screening tool to detect early stages of pancreas cancer, it was observed that in the 75% of breast cancer patients the levels of circulating EXOs positive for GPC1 were higher than in healthy individuals; the data on breast cancer, though, did not support any specific correlation between the level of EXOs positive for GPC1 and breast cancer subtypes, maybe for a size too small of the observed group [117]. Fibronectin associated with the EV surface was also suggested as a promising marker to detect breast cancer [135].

Recently, it was reported the role of exosomal protein CD82 as a diagnostic biomarker for precision medicine in BC. CD82 is a member of tetraspanin superfamily that exert its activity via tetra-transmembrane protein enriched microdomains in EXOs. In particular, it was demonstrated that CD82 expression in BC tissue was significantly lower than in healthy and benign breast disease tissues. There was a negative correlation between CD82 expression in tissues and CD82 content in EXOs, which indicated that CD82 expression was redistributed from tissues to the blood with the development and metastasis of BC [136].

It is evident that the potential of EVs as biomarkers for prognosis or response to therapy in patients with tumors is still actively being investigated [96,112,113,114,137,138]. Innovative findings encourage the researchers to also study the potential role of EVs isolated from the blood of patients undergoing immunomodulatory cancer therapies [83].

In addition to in vitro and in vivo studies, many clinical trials are also ongoing to understand the actual application of EVs as biomarkers; among these trials, many are related to BC [106,139].

Even if clinical opportunities for EVs use as biomarkers are appealing it must not be forgotten that many potential challenges still affect this field. Among these, the first one is the need to identify the appropriate candidate as reliable clinical biomarker in a huge pool of molecules. It is also necessary to understand which is the most useful biological fluid for each single pathology, not least because it will be necessary to develop suitable technologies for routine analysis that require small volumes, are rapid, sensitive, and specific, and can be automated. The techniques today used in the EVs study rarely meet these requirements, thus a great technological effort will be necessary to reach the goal of EVs use in clinical settings [106].

Nowadays, concerning the EVs clinical applications, it is important to mention that great attention is paid to their potential use as “shuttles” for targeted therapies in cancer treatment. EVs, indeed, could be engineered to deliver specific biomolecules to specific target cancer cells. This approach has been taken in consideration because, today, there is a great effort in trying to make therapeutic treatments able to selectively reach the tumor, so as to avoid systemic adverse effects. Cancer patients, in fact, often require prolonged drugs’ administration over time, which leads to the achievement of high doses but also causes several systemic adverse effects. Many possible synthetic delivery systems have been considered from this point of view including liposomes, micelles, nano-capsules and nanospheres. Each of these systems has their own advantages and disadvantages, as well as peculiar features in terms of structure, molecular composition, and drug release rate. To them, more recently, EVs have been added [140,141].

A multitude of studies are exploring the possibility of using EVs as vehicles for drug delivery, taking into due consideration all the aspects and steps that may affect their clinical functionality, such as the most appropriate cell source, the isolation methods, the loading mechanisms, the approaches to obtain an efficient and specific targeting, and the best route of administration [140,141].

The bisphosphonates are clinically used to treat bone complications of tumors. However, bisphosphonates act only on mature, actively resorting osteoclasts, but have no effect on residual osteoclasts. This might explain why zoledronic acid, approved by Food and Drug Administration (FDA) to treat patients with solid tumor bone metastasis, does not affect survival rate of the patients. Nowadays, RANKL/RANK system-based therapeutic strategies are also used to treat BC bone metastasis. Since RANKL plays a key role in the progression of primary breast cancer and bone metastasis, targeting RANKL is an important therapeutic approach for patients with osteoclastic lesions. Moreover, as OPG is one of the receptors of RANKL, interfering with RANKL function might be an efficient way of decreasing osteolytic lesions. For this reason, a genetically modified recombinant OPG-Fc construct (AMGN-0007) was developed for treating patients with bone metastases. Nevertheless, the short half-lives of this kind factors have prevented further research and application of this molecules in clinical management [18]. Engineered EVs could be used to convey and improve the half-lives of these innovative molecules. Furthermore, BC cells can induce osteoclasts activation via EXOs, as described in multiple myeloma [89] and NSCLC [90]. The modulated release of EVs by BC cells or EVs uptake by target cells should represent a new therapeutic strategy to counteract the metastasis and restore the normal bone remodeling. The advantages of cancer-derived EVs over other synthetic system include that they should bear tumor-specific antigens that can prime immune cells to induce an immune response and that they are naturally produced by the cells, so they should interact more “physiologically” with biological targets, also ensuring unique homing features and tissue tropism [142]. In an in vivo study on a mouse model, it has even been shown that properly selected EVs, once injected intravenously, are specifically able to reach neurons, microglia, and oligodendrocytes in the brain (thus crossing the blood brain barrier) and to efficiently convey the loaded siRNA supporting their use for brain-targeted delivery [143].

The results obtained so far are encouraging, but many studies are required to ensure that EVs may truly represent an opportunity as a delivery system and to pave the way for development of EV-based cancer therapies.

## 7. Conclusions

The principal aim of cancer research is the complete comprehension of metastatic progression and its dynamics. The colonization of secondary organs by cancer cells does occur in an organ-specific manner, depending on the cancer type. EVs constitute a bi-directional interaction, mediating the crosstalk between cancer cells and their microenvironment to promote tumorigenesis, cancer progression, metastasis, and drug resistance. Although EVs are still under-exploited in the clinical setting, the studies reported in this review demonstrate the multiple roles of EVs in breast cancer and their possible use in the development of future therapies for breast cancer patients.

The discovery of early biomarkers in human fluids, with minimally invasive and low-cost methods, could support novel therapeutic approaches. In the era of liquid biopsy, the use of EVs or proteins or miRNAs contained in them could improve clinical practice, favoring an increasingly timely diagnosis and better management of breast cancer and bone metastasis.

## Figures and Tables

**Table 1:** EV-associated miRNAs for which a role in metastasis has been reported for BC.

EVs-Associated miRNAs	Biological Role	Molecular Mechanism	Reference
miR-222/223	Quiescence induction	Not described	[96]
miR-23b	Cell dormancy induction	Suppresses MARCKS gene, encoding protein that promotes cell cycling and motility	[95]
miR-105	Endothelium integrity weakening	Targets ZO-1, a tight junction protein	[99]
miR-122	Energy metabolism reprogramming	Downregulates the glycolytic enzyme pyruvate kinase	[102]
MiR-940	Osteogenic differentiation induction	Targets ARHGAP1(a GTPase-activating proteins) and FAM135A (has roles in tumor metastasis promotion)	[92]

## Abbreviations

ADAM12	Metalloproteinase domain-containing protein 12
AREG	Amphiregulin
BC	Breast Cancer
BMM	Bone Marrow-derived Mononuclear
BRCA	BReast CAncer gene
BSA	Bovine Serum Albumin
CAF	Cancer-associated Fibroblasts
CASP8	caspase 8
CCL3	Chemokine (C-C motif) ligand 3
circRNA	circularRNA
CTCs	Cancer Tumor Cells
ctDNA	circulating tumor DNA
ctRNA	circulating tumor RNA
CXCR4	C-X-C chemokine receptor type 4
EMT	Epithelial–Mesenchymal Transition
ERBB2	erb-b2 receptor tyrosine kinase 2
EVs	Extracellular Vesicles
EXOs	Exosomes
IGF	Insuline-like Growth Factor
lncRNA	long non coding RNA
miRNA	microRNA
MVs	Microvesicles
NSCLC	Non-Small Cell Lung Cancer
OPG	Osteoprotegerin
PAI	Plasminogen activator inhibitor type 1
PARP1	Poly ADP-Ribose Polymerase 1
PARP2	Poly ADP-Ribose Polymerase 2
PDGF	Platelet Derived Growth Factor
PTHrP	Parathyroid hormone-related protein
RANKL	Receptor-Activator-of-Nuclear-factor-Kappa-B-Ligand
SNPs	Single Nucleotide Polymorphisms
SOCE	Store-Operated Calcium Channel
TEP	Tumor educated platelets
TGFβ	Transforming Growth Factor β
TNBC	triple-negative breast cancer
TRPs	Transient Receptor Potential cannels
Tspan8	Tetraspanin 8
VGCCs	Voltage-Gated Calcium Channels
